# A Universal Vaccine against Leptospirosis: Are We Going in the Right Direction?

**DOI:** 10.3389/fimmu.2017.00256

**Published:** 2017-03-09

**Authors:** André Alex Grassmann, Jéssica Dias Souza, Alan John Alexander McBride

**Affiliations:** ^1^Biotechnology Unit, Technological Development Centre, Federal University of Pelotas, Pelotas, Brazil; ^2^Gonçalo Moniz Institute, Oswaldo Cruz Foundation, Ministry of Health, Salvador, Brazil

**Keywords:** *Leptospira*, reverse vaccinology, genome mining, vaccine discovery, vaccine candidate, recombinant vaccine, subunit vaccine, animal model

## Abstract

Leptospirosis is the most widespread zoonosis in the world and a neglected tropical disease estimated to cause severe infection in more than one million people worldwide every year that can be combated by effective immunization. However, no significant progress has been made on the leptospirosis vaccine since the advent of bacterins over 100 years. Although protective against lethal infection, particularly in animals, bacterin-induced immunity is considered short term, serovar restricted, and the vaccine can cause serious side effects. The urgent need for a new vaccine has motivated several research groups to evaluate the protective immune response induced by recombinant vaccines. Significant protection has been reported with several promising outer membrane proteins, including LipL32 and the leptospiral immunoglobulin-like proteins. However, efficacy was variable and failed to induce a cross-protective response or sterile immunity among vaccinated animals. As hundreds of draft genomes of all known *Leptospira* species are now available, this should aid novel target discovery through reverse vaccinology (RV) and pangenomic studies. The identification of surface-exposed vaccine candidates that are highly conserved among infectious *Leptospira* spp. is a requirement for the development of a cross-protective universal vaccine. However, the lack of immune correlates is a major drawback to the application of RV to *Leptospira* genomes. In addition, as the protective immune response against leptospirosis is not fully understood, the rational use of adjuvants tends to be a process of trial and error. In this perspective, we discuss current advances, the pitfalls, and possible solutions for the development of a universal leptospirosis vaccine.

## Introduction

Following the discovery of leptospirosis, it was primarily associated with rural populations (1). This disease is caused by pathogenic *Leptospira* spp. and can be transmitted by direct contact via infected animals or by indirect contact as leptospires can survive outside the host. Agricultural workers, mineworkers, veterinarians, or individuals that came into direct contact with infected animals or contaminated environments were the main at-risk groups. However, toward the end of the 20th century, there were reports of leptospirosis among the homeless in major cities in the USA (2) and in urban slum communities in developing countries (3). The WHO estimated that the global incidence of leptospirosis more than doubled from approximately 500,000 cases in 1999 (4), to over a million cases in 2015 (5). Urban leptospirosis is now endemic in urban slums due to the lack of sanitation, rodent infestation, extreme poverty, and limited access to public health services in these communities.

Severe leptospirosis or Weil’s disease (jaundice, acute renal failure, and bleeding) has a case fatality rate of >10%. However, leptospirosis-associated pulmonary hemorrhage syndrome (LPHS) is being increasingly reported in developing countries (6) and the fatality rate is >50% (7). Clinical diagnosis of leptospirosis is difficult due to its similarity with other hemorrhagic diseases, and laboratory diagnostic tests are inadequate in these settings (8). There remains an urgent need for point-of-care rapid diagnostic tests. Vaccination of at-risk populations remains the most viable strategy for the control of leptospirosis. Classical, inactivated, vaccines have been available for over 100 years and are used routinely for agricultural and companion animals, reviewed in Ref. (9). Some countries have approved their use in human at-risk populations, although due to the severe side-effects and perceived short-term immunity and lack of cross-protection, they have not been adopted by the global community (10).

Of the 22 known *Leptospira* spp., 15 are infectious and can cause disease with varying degrees of severity. The pathogenesis of leptospirosis is a multifactorial process that is poorly understood, see, e.g., Ref. (11). Serological classification of leptospires indicates the existence of at least 250 serovars distributed in 18 serogroups (12). All this genetic and phenotypic diversity of pathogenic *Leptospira* spp. is a major drawback for vaccine development. The idea of a universal vaccine capable of protecting against all infectious *Leptospira* spp. and serovars would appear to be farfetched. However, some progress has been made with other pathogens such as influenza (13, 14), dengue (15), and others (16, 17). This perspective focuses on current advances, limitations, possible solutions, and looks forward to the possibility of a universal leptospirosis vaccine.

## Experimental Recombinant Vaccines

LipL32 is the immunodominant protein in pathogenic *Leptospira* spp. (18), there are over 38,000 copies per cell (19), and it can comprise up to 75% of the protein content of the outer membrane (OM) (20). However, there is a doubt as to its cellular localization; the latest report suggests it may occupy a subcellular location on the inner leaflet of the OM (21). LipL32 is not required for virulence; an *Leptospira interrogans lipL32* knockout mutant could still infect hamsters (22). The biological function of LipL32 remains unknown, yet it is remarkable that such an abundant protein can be removed from the leptospiral OM with little or no effect on growth rate or OM structure. This is an example of the redundancy encoded in the *Leptospira* genome, as seen with other proteins, e.g., putative adhesins (23). There are over 20 publications on LipL32 and vaccine development. However, when rigorous statistical analysis is applied [e.g., Fisher’s exact test (24)], only five demonstrated significant protection against leptospirosis, reviewed in Ref. (10, 25). In addition, problems with reproducibility, survival in the control groups, high challenge doses (septic shock or leptospirosis), and the subcellular location of LipL32 have complicated its candidacy for inclusion in a universal vaccine formulation.

The leptospiral immunoglobulin-like (Lig) protein family includes LigA, LigB, and LigC and is only found in pathogenic *Leptospira* spp. (26, 27). While LigA and LigB are highly conserved (28), only LigB is present in all pathogenic *Leptospira* spp. (29). LigA and LigB are virulence determinants that are upregulated during infection (30), play a role in host cell adherence (31), prevent blood clotting (32, 33), and inhibit complement (34, 35). However, as seen for LipL32, an *L. interrogans ligB* knockout mutant remained virulent in the hamster model (36). Nevertheless, the Lig proteins are the standout vaccine candidates to date, with high, reproducible, levels of protection in animal models of acute leptospirosis in over 15 scientific reports, although not all withstood rigorous statistical analysis (10). The C-terminal (non-identical) region of LigA is an accepted vaccine candidate, having been evaluated in subunit (37–41), DNA (42), encapsulated (43), lipidated (44), and carbon nanotube vaccine preparations (45). However, when evaluated in a hamster colonization model, LigA failed to prevent infection (46). There is less evidence in support of LigB, the N-terminal conserved (repeat) region conferred significant protection as a subunit vaccine preparation (47) and a DNA vaccine (48) in the hamster model. Our group found that the same LigB polypeptide (LigBrep) not only protected hamsters but also induced sterile immunity in survivors (manuscript submitted).

Using the classical approach to vaccine candidate discovery, approximately 30 leptospiral, non-LipL32, non-Lig, proteins have been evaluated (10, 25). Of these, 10 proteins conferred significant protection against challenge with *Leptospira* spp. when the data were reanalyzed using, when necessary, a more rigorous statistical analysis (Fisher’s exact test) (10). The first report of protein-based protection came from studies of recombinant OmpL1 and LipL41 in the hamster model (49), and although only 1/3 experiments demonstrated significant protection, this provided the initial impetus for further research into protein-based vaccine candidates against leptospirosis. In an evaluation of three putative OMPs (Lp1454, Lp1118, and MceII), the subunit formulations failed to protect hamsters (50); however, when combined and encapsulated in liposomes, they conferred significant protection against challenge (51, 52). The putative lipoprotein LemA, identified using a reverse vaccinology (RV) approach (53), significantly protected immunized hamsters when administered as a DNA vaccine and protection increased using a prime-boost strategy (*lemA*/LemA) (54). In the most extensive study to date, 238 proteins identified using RV were evaluated as vaccine candidates (55). A hamster colonization model was used to evaluate pools of recombinant proteins (5 proteins/pool) and >70% were immunogenic. However, none of the recombinant protein pools conferred protection against colonization.

## Target Discovery

Cytoplasmic proteins, inner membrane proteins, and OM lipoproteins that are not exposed on the surface (i.e., those attached to the inner leaflet of the OM) are likely to be ineffective recombinant vaccines. Antibodies induced by subsurface proteins would not be able to bind to infecting leptospires making the vaccine ineffective. Therefore, vaccine candidates should be surface exposed on the leptospiral cell. Equally important are the potential roles in pathogenicity and the immunogenicity of these proteins. Furthermore, it is doubtful that a protein-based vaccine candidate would be capable of inducing a protective immune response if the protein components of the vaccine lacked one or more of these characteristics.

**Figure 1:**
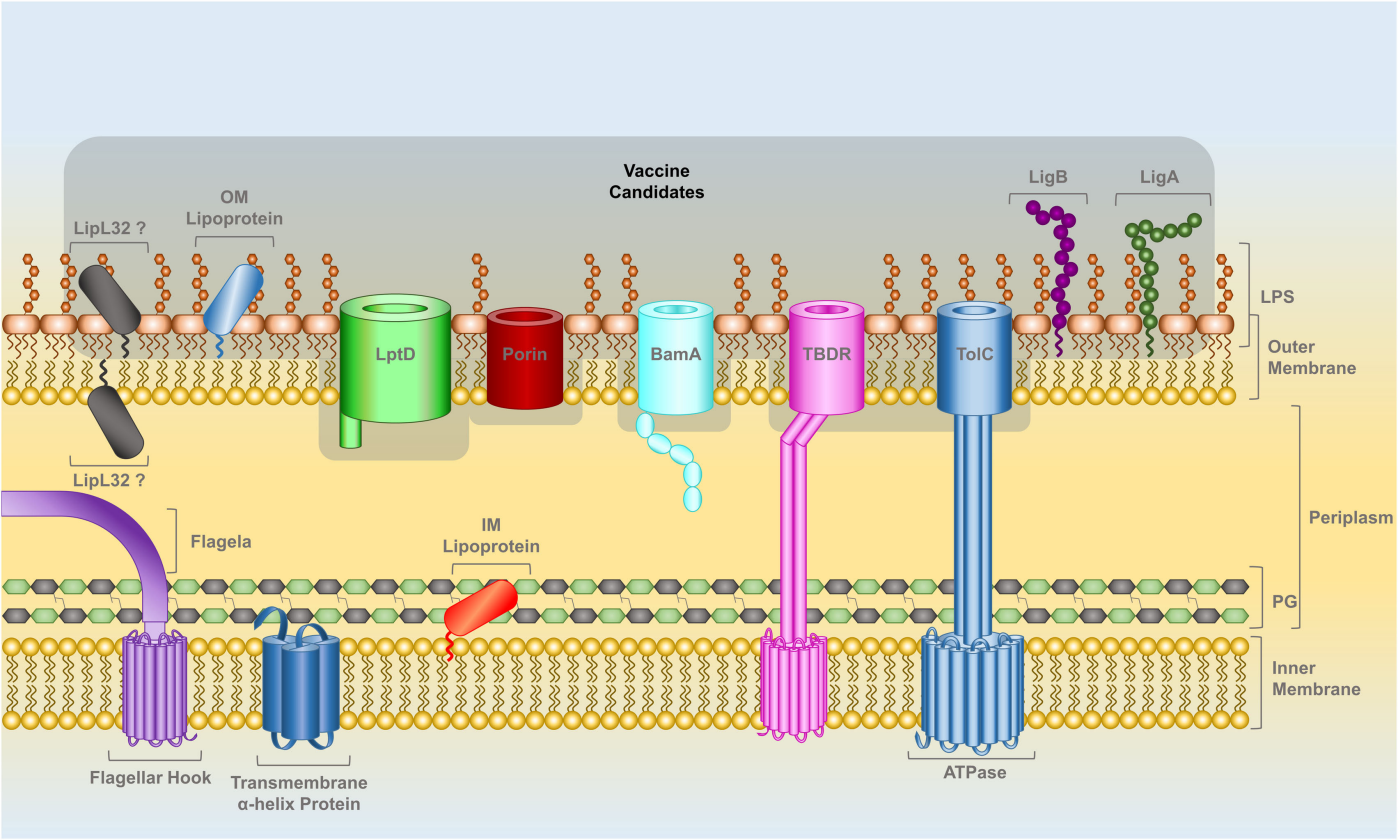
**The cell wall of *Leptospira* spp., a diderm bacteria with inner and outer lipid membranes, is the target for the identification of potential vaccine candidates**. The outer leaflet of the outer membrane (OM) is composed of lipopolysaccharide. Lipoproteins can be attached by a lipid anchor to one of the leaflets of either membranes. The IM is spanned by alpha-helix transmembrane proteins while beta-barrel transmembrane proteins span the OM. Leptospiral motility is provided by two flagella that are attached to the IM and are located within the periplasmic space (PS). A peptidoglycan layer is also present in the PS. OM lipoproteins, such as LigA and LigB, as well as OM beta-barrel proteins, such as LptD, BamA, TolC-, TonB-dependent receptors, and other porins have at least a portion of their structure exposed on leptospiral surface and are prospective vaccine candidates, highlighted in gray. The localization of the lipoprotein LipL32 in the OM is controversial; the latest reports indicate that it has a subsurface location (see text).

Lipoproteins attached to the outer leaflet of the OM and transmembrane β-barrel proteins spanning the OM (OMPs) should be fully or partially surface exposed (Figure 1). The localization of LipL32 is still unresolved; there is experimental data for both surface (56–59) and subsurface locations (21). Leptospiral genomes encode OMPs such as LptD, BamA-like, TonB-dependent receptors, and several other porins that play crucial roles in bacterial survival and potential role in pathogenicity. These proteins are ideal targets and should be evaluated as potential vaccine candidates. RV was developed to identify surface-related proteins in the genome of pathogens using bioinformatics (60). RV has been used to analyze *Leptospira* genomes and there are several reports in the literature that have used *in silico* genome mining toward the identification of leptospiral vaccine candidates, reviewed in Ref. (61).

Recently, dozens of leptospiral proteins have been described as adhesins, reviewed in Ref. (11), and blocking the adhesion of leptospires is thought to impair their virulence. Similarly, several proteins have been described as host complement activation inhibitors, suggesting that leptospires evade the complement system, reviewed in Ref. (62). In many studies (63–67), the surface localization of the leptospiral antigens were determined by *in vitro* approaches including proteinase K digestion and a surface immuno-fluorescence assay (IFA) (68). These approaches have contributed to the controversy surrounding the localization of proteins such as LipL32. Another example is that of LIC13166, this protein was originally demonstrated to be an OMP exposed on the surface of the leptospiral cell by surface biotinylation, membrane affinity, and surface-IFA experiments (68). However, in a recent publication, it was shown that LIC13166 is, in fact, a flagellar protein, renamed FcpA, which is located in the periplasm (69). The subcellular location of adhesins, complement binding proteins, and virulence factors described in knockout experiments should be properly investigated; otherwise the biological relevance of these findings will remain unclear. We are currently developing an alternative method to improve the identification of surface leptospiral proteins while maintaining the integrity of the leptospiral OM.

## Cross-Protection

The perceived lack of cross-protection following immunization with a bacterin is another factor that has limited their widespread use. There are, however, several reports of bacterins conferring cross-protection against species-related serovars. An evaluation of bacterins reported 100% cross protection between *L. interrogans* serovars Canicola, Copenhageni, and *Leptospira borgpetersenii* serovar Ballum but not serovar Mozdok (70). A multivalent bacterin containing serovars from four different serogroups demonstrated cross-protection in a canine model of leptospirosis (71). Another study of two bacterins based on different serovars, but belonging to the same serogroup and species, reported species-related cross-protection, although 50% of the control group survived (72). It is likely that the protective antigens in these studies were proteins, as leptospiral lipopolysaccharide (LPS) does not induce cross-protection, even among species-related serovars (73). Rather, protein-enriched samples were responsible for cross-protection against species-related serovars in a gerbil model of lethal leptospirosis. This was further supported by a study using a live vaccine based on an LPS defective mutant. Species-related cross-protection was demonstrated, although the vaccine could not prevent colonization by a non-related serovar (74). Several studies of individual proteins have claimed to show cross-protection. An adenovirus construct containing *lipL32* conferred cross-protection against a species-related serovar, although >50% of the control groups survived (75). A treatment based on anti-LipL32 monoclonal antibodies protected hamsters challenged with a species-related serovar (76). Prime-boost strategies using LemA and LigBrep conferred cross-protection against a species-related serovar, albeit in one-off experiments (54, 77).

**Figure 2:**
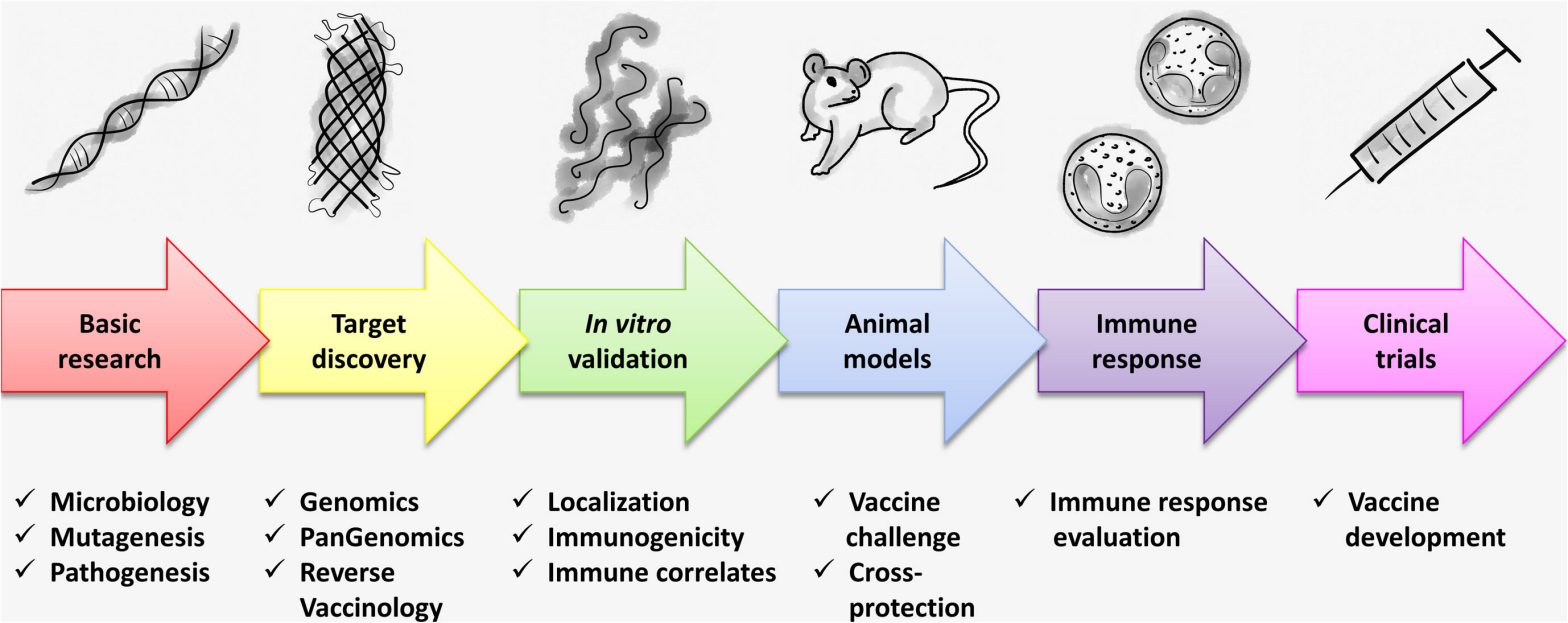
**A schematic representation of the development pipeline for a universal vaccine against leptospirosis**. The basic research on *Leptospira* microbiology and molecular biology contributes to vaccine development. *Leptospira* mutagenesis is an example of basic research that has and will continue to further our understanding of pathogenesis and identification of virulence factors. Genomic and pangenomic studies are of central importance to the development of a universal leptospirosis vaccines, permitting the identification of potential vaccine candidates and the analysis of protein sequences among different *Leptospira* spp. RV has not been fully explored in leptospirosis and needs to be more thoroughly exploited. Once potential vaccine candidates are identified, an *in vitro* validation is required, particularly to confirm the localization of antigens on the surface of the leptospiral cell. At this stage, a prospective vaccine candidate can be assessed for immunogenicity. The lack of well-defined correlates of immunity for leptospirosis represents one for the major limitations for leptospirosis vaccine development and remains to be resolved. Therefore, surface-related, conserved (among infectious *Leptospira* spp.), and immunogenic leptospiral antigens must be evaluated in vaccine challenge experiments using animal models. Cross-protection, defined as cross-species rather than cross-serovar protection should be evaluated. In addition, as the protective immune response is not fully understood, continued research in this field is necessary. Finally, the long-term goal of this pipeline is to identify experimental vaccine preparations for evaluation in clinical trials.

At least one strain for every known *Leptospira* spp. has been sequenced and new isolates are continually being sequenced and their genomes released on GenBank or other public databases, see, e.g., Ref. (29, 78–83), thereby providing a panoramic view of *Leptospira* pathogenomics, permitting the identification of orthologs and protein sequence similarity among infectious species. This has significantly contributed to the identification and selection of conserved vaccine candidates based on a simple *in silico* sequence analysis (Figure 2). Protein sequences are usually highly conserved among the same species regardless of the serovar or serogroup, while they can differ considerably when comparing the same serovar in different species. While, serological classification is unquestionably important for epidemiology and bacterin-based vaccine studies, it is of limited use for recombinant vaccine development. This is a problem associated with a leptospiral bacterin vaccine, the immune response is primarily directed against the leptospiral LPS and while it protects against infection by closely related serovars or serovars from the same *Leptospira* spp., leptospiral LPS does not stimulate memory B-cells (10). As there is no clear definition of cross-protection in the field of leptospirosis, this is a major drawback to vaccine candidate discovery and evaluation. Ideally, recombinant vaccine-induced cross-protection should be defined as cross-species protection rather than cross-serovar protection. A universal vaccine should therefore protect against all 15 infectious *Leptospira* spp. regardless of serovar. However, if this is not a viable option, it should be possible to identify the main circulating species and develop a region-specific recombinant vaccine rather than a universal vaccine. This could potentially allow the characterization of the protective immune response and establish standard protocols for the evaluation of cross-protection of recombinant vaccine candidates (Figure 2).

## Modulation of the Immune Response

Several adjuvants and delivery systems have been used to enhance the immune response against leptospiral antigens. Aluminum hydroxide (alhydrogel) and Freund’s adjuvant are by far the most common, although others including flagellin (84), CpGs (85), nanostructures (45), liposomes (43, 51, 52), xanthan (85) have been investigated. While Freund’s adjuvant cannot be used in humans due to its high reactogenicity (86), it is the most potent commercially available adjuvant (87), is useful for the primary screening of vaccine antigens, and has been used successfully in vaccine formulations against leptospirosis (39, 41). To date, only partial protection has been demonstrated with vaccines using alhydrogel, the most widely used adjuvant in human vaccines. Recently, other adjuvants have become commercially available and have been approved for use in the formulation of human vaccines, comprising the adjuvants MF59 (squalene), AS01 [monophosphoryl lipid A (MPL), QS21], AS03 (α-tocopherol, squalene, and polysorbate 80), AS04 (MPL combined with alhydrogel), and virosomes (liposome/VLPs) (88, 89). These prospective adjuvants have not yet been evaluated as adjuvants for leptospirosis vaccines.

Rational modulation of the immune response is difficult to achieve for leptospirosis vaccines as little is known about the protective immune response that should be induced by a leptospirosis vaccine. Humoral immunity is believed to be responsible for protection; anti-LPS antibodies are protective in animal models and can be passively transferred between animals (90). As predominantly extracellular organisms, leptospires are most likely cleared from the bloodstream by phagocytosis followed by opsonization. However, at least in some hosts, e.g., cattle, induction of cellular immunity is equally important (90). Until recently, there were no published reports of correlation between antibody titer, induced by leptospiral recombinant vaccines and protection against challenge. However, an oral immunization strategy based on LigA found that survival was dependent on a minimum antibody titer being reached in a 2-week period following immunization (44), and if this can be reproduced, it will be an extremely important finding. The lack of immune correlates is a major limitation in target discovery using RV as they are essential for the *in vitro* screening of potential vaccine candidates, see, e.g., the bactericidal assay for *Neisseria meningitidis* (91) and the opsonophagocytosis assay for *Staphylococcus aureus* (92).

## Animal Models of Leptospirosis

The recommended animal model for acute leptospirosis is the Syrian hamster; this model replicates the human symptoms of the disease, including kidney failure, LPHS, and kidney, liver, and lung tissue damage, which result in death (93). Furthermore, the hamster model is the recommended model for potency testing of bacterin vaccines (94). The acute model is dependent on a virulent challenge strain and the lack thereof has had a major impact on protection studies. However, to date, no well-established correlates of immunity have been identified and, therefore, vaccinated hamsters must be challenged with a virulent *Leptospira* strain to demonstrate protection. Due to significant variation among the hamster models of acute leptospirosis, we recommend that the research community adopt a standardized model (see Supplementary Material). An alternative to the lethal model is the hamster colonization model, and this is the model of choice when evaluating vaccine candidates for agricultural animals including cattle, swine, and horses (55, 95). Unlike the acute model, the primary endpoint in this model is kidney colonization.

A major limitation of the hamster model is the lack of commercial reagents for characterization of the immune response, e.g., induction of cytokines and chemokines cannot be measured directly. Alternate models include the guinea pig and the gerbil, although there are few commercially available reagents for these models. Due to the wide range of commercially available reagents, the mouse model is attractive, reviewed in Ref. (96). Wild-type mice are naturally resistant to leptospirosis, although colonization is possible with some serovars (9, 97). Lethal leptospirosis has been demonstrated in C3H/HeJ (41), SCID, and Rag1 knockout mice (98). Maintenance host models of chronic infection have been developed using the Wistar strain of *Rattus norvegicus* (9, 99).

## Conclusion

Alternatives to whole-cell inactivated leptospiral vaccines have so far failed to live up to their initial promise, and the concept of a universal leptospiral vaccine remains just that, a concept. Several reviews have highlighted the modest numbers (~30) of leptospiral proteins that have been tested using various vaccine strategies, including subunit, DNA vaccines, prime-boost, encapsulated, and live avirulent strains. Of these, less than a handful has been successful. However, the availability of multiple genome sequences, combined with advances in bioinformatics (e.g., RV) and the characterization of surface-exposed virulence factors, will improve the discovery of potential vaccine candidates. The next challenge is to develop *in vitro* assays based on correlates of immunity for the high-throughput screening of these vaccine candidates. While there are several animal models of leptospirosis, their standardization is necessary for the critical interpretation of protection data. Cross-protection is a priority for a universal vaccine and will require the identification of vaccine candidates that are conserved among the infectious *Leptospira* spp. Our poor understanding of the (protective) immune response has hindered the intelligent selection of adjuvants for use in vaccine formulations. Finally, while the field is moving in the right direction, a universal vaccine for leptospirosis remains a long-term goal.

## Author Contributions

AG, JS, and AM wrote the manuscript. AG and JS created the figures, and all the authors contributed to and revised the manuscript.

## Conflict of Interest Statement

AM is an inventor on several patents for the use of *Leptospira* proteins as vaccines and diagnostics. The other authors declare no conflict of interest.
