# Supplementary material for: A Universal Vaccine against Leptospirosis: Are We Going in the Right Direction?
Source: Front Immunol. 2017 Mar 9;8:256. doi: 10.3389/fimmu.2017.00256 (PMC5343615; doi:10.3389/fimmu.2017.00256)
Supplement: Supplementary file 1 [file Data_Sheet_1.DOCX]

Supplementary Material

**A universal vaccine against leptospirosis: Are we going in the right direction?**

**André Alex Grassmann^1^, Jéssica Dias Souza^1^, Alan John Alexander McBride^1,2*^**

*** Correspondence:** Dr. Alan McBride, alan.mcbride@ufpel.edu.br

# Supplementary Data

**Proposal of a standard protocol for experimental vaccine evaluation in the hamster model of acute leptospirosis**

**Animals:**

All animals should be treated according to local and international ethics in animal experimentation, obtaining approval from ethics committees according to local legislation. Syrian Hamsters (*Mesocricetus auratus*) aging 4-6 weeks-old, maintained in an authorized animal house, with access to food and water *ad libitum*, under biosafety level 2 biocontainment and according to local legislation. Ideally, the animals should be specific pathogen free.

**Experimental design:**

The number of animals per experimental group should be determined using a power calculation, see e.g. http://hedwig.mgh.harvard.edu/sample_size/fisher/js/fisher.html, and is dependent on the statistical analysis that will be used to determine the significance of protection. We recommend groups of 8-10 animals/group, based on 10% survival in the control group and 90% protection in the vaccinated group. There will be at least 93.2% chance of detecting a significant difference between the groups (n=8/group), based on *P* < 0.05. Fisher’s exact test (two-tailed) is based on a 2×2 contingency table (1), and is recommended for the evaluation of protection against death in vaccine experiments (2). The use of a Log-Rank statistical analysis is not recommended as it is more suited to evaluating increased survival between groups rather than protection against death (3).

A sham-immunized group should be included with at least the same number of animals as the vaccine groups. Usually this group receives saline in the same volume and via as the vaccine group. Alternatively, the negative control group can be injected with adjuvant alone. Both saline and adjuvant only groups can be used in the same experiment. The use of a bacterin group is unnecessary unless it is crucial for the hypothesis being tested. There is overwhelming evidence that bacterins are protective against lethal leptospirosis in a homologous challenge. In addition, this will adhere to the principles of the 3Rs by reducing the unnecessary use of animals.

**Vaccine preparation and immunization**

The dosage, frequency of booster doses and the route of immunization will depend on the vaccine strategy. For subunit vaccines, we recommend the use of 50-100 µg of protein per dose, in a volume ≤ 200 µl per animal (dependent on animal age and legislation), two to three doses, with 2-3 weeks between each immunization.

**Vaccine challenge**

A virulent *Leptospira* strain is necessary to establish acute leptospirosis in the control groups. LD_50_ experiments should be designed (when allowed by local ethics committees) respecting the 3Rs principle, with 3-4 animals per group, evaluating smaller challenge doses (e.g. 10^0^, 10^1^, 10^2^, 10^3^) first and in the case of absence of virulence, higher doses could be evaluated in a second experiment. The vaccine challenge doses should be high enough to reach the endpoint criteria in 100% of the sham-immunized group. The challenge dose that resulted in the establishment of lethal leptospirosis in 100% of the negative control groups reported in literature varies from 2.5 to 200 × LD_50_. The number of leptospires inoculated during challenge usually varies from 10^2^ to 10^4^ per animal. Higher challenge doses are indicative of loss of virulence and can cause septic shock rather than leptospirosis in the infected animals.

The standard challenge route is the intra-peritoneal (IP) inoculation of leptospires in EMJH or PBS (≤ 1 ml). However, alternative methods of infection include conjunctival, intradermal inoculation and transcutaneous infection (personal communication, manuscript in preparation) that mimic the natural route of infection.

Survival of animals in the control groups is one of the most common problems reported for leptospirosis vaccine experiments, see (2). This problem can be reduced by using the correct long-term storage conditions, liquid nitrogen rather than ultrafreezers, low-passage cultures (< 3 passages *in vitro*) for the challenge dose and the use of a leptospiral culture at a standardized phase of the *in vitro* growth. Virulence can sometimes be recovered by infection of hamsters and recovery of a virulent isolate from the kidneys of infected animals. Seed lots of the virulent strain recovered from hamster kidney should be expanded and stored long-term in liquid nitrogen (4).

**Endpoints**

The primary endpoint is death and as it is unethical for a laboratory animal to suffer until this endpoint is reached, several secondary markers can be used to predict death. These include weight loss (> 10%) and changes in behavior such as lack of preening, isolation, failure to respond to stimulus (5, 6). Animals should be monitored three times per day until clinical signs of leptospirosis are detected. From this point, the animals should be monitored every two hours until endpoint criteria for euthanasia are met. The clinical signs of death should be seen from the eighth or ninth day onwards post-challenge, if clinical signs are seen after 5 days or less this is indicative of septic shock and not leptospirosis.

**Supplementary References:**

1. Fisher RA. *The Design of Experiments*: Macmillan (1935).

2. Adler B. Vaccines against leptospirosis. *Current topics in microbiology and immunology* (2015) **387**:251-72. doi: 10.1007/978-3-662-45059-8_10. PubMed PMID: 25388138.

3. Mantel N. Evaluation of survival data and two new rank order statistics arising in its consideration. *Cancer Chemother Rep* (1966) **50**(3):163-70. PubMed PMID: 5910392.

4. WHO, ILS. *Human leptospirosis: guidance for diagnosis, surveillance and control.* Malta: World Health Organization (2003).

5. Coutinho ML, Choy HA, Kelley MM, Matsunaga J, Babbitt JT, Lewis MS, et al. A LigA three-domain region protects hamsters from lethal infection by Leptospira interrogans. *PLoS Negl Trop Dis* (2011) **5**(12):e1422. Epub 2011/12/20. doi: 10.1371/journal.pntd.0001422

PNTD-D-11-00830 [pii]. PubMed PMID: 22180800; PubMed Central PMCID: PMC3236721.

6. Humphryes PC, Weeks ME, AbuOun M, Thomson G, Nunez A, Coldham NG. Vaccination with leptospiral outer membrane lipoprotein LipL32 reduces kidney invasion of Leptospira interrogans serovar canicola in hamsters. *Clinical and vaccine immunology : CVI* (2014) **21**(4):546-51. doi: 10.1128/CVI.00719-13. PubMed PMID: 24521782; PubMed Central PMCID: PMCPMC3993109.
